# Eplerenone Inhibits Atrial Autonomic Nerve Remodeling in Atrial Fibrillation Through ERK1/2 MAPK Pathway

**DOI:** 10.1155/cdr/6041636

**Published:** 2025-07-28

**Authors:** Wei Xu, Cheng-yuan Yu, Ding-yu Wang, Qiang Gao, Song Zhang, Yun Zhang, Yue Yuan, Jing Shi, Yue Li, Guang-zhong Liu, Xiao-ming Shang

**Affiliations:** ^1^Department of Emergency Medicine, The First Affiliated Hospital, Harbin Medical University, Harbin, Heilongjiang, China; ^2^Department of Geriatrics, Shenzhen People's Hospital (The First Affiliated Hospital, Southern University of Science and Technology; The Second Clinical Medical College, Jinan University), Guangdong Provincial Clinical Research Center for Geriatrics, Shenzhen Clinical Research Center for Geriatrics, Shenzhen, Guangdong, China; ^3^Department of Cardiology, The First Affiliated Hospital, Harbin Medical University, Harbin, Heilongjiang, China; ^4^Department of Cardiology, Shenzhen People's Hospital (The First Affiliated Hospital, Southern University of Science and Technology; The Second Clinical Medical College, Jinan University), Guangdong Provincial Clinical Research Center for Geriatrics, Shenzhen Clinical Research Center for Geriatrics, Shenzhen, Guangdong, China; ^5^Department of Cardiology, Shenzhen Luohu District Hospital of Traditional Chinese Medicine, Shenzhen, Guangdong, China

## Abstract

Atrial autonomic nerve system (ANS) remodeling plays an important role in atrial fibrillation (AF). Mineralocorticoid receptor antagonists (MRAs) have been proved to be effective in preventing atrial structural remodeling. However, the effects of MRA on ANS remodeling in AF and the underlying mechanisms are still unknown.

**Methods:** Then, 21 rabbits were randomized into sham, pacing, and pacing + eplerenone groups. To verify the effect of aldosterone on ANS remodeling, 18 SD rats were pumped with aldosterone. HL-1 cells were subjected to control treatment or rapid pacing with or without eplerenone or U0126 (an inhibitor of ERK1/2). Atrial sympathetic and parasympathetic remodeling was detected by immunohistochemical staining, Western blotting, and RT-PCR. The circulating neurohormone and atrial electrophysiology were also assessed.

**Results:** The ERK1/2 MAPK pathway was significantly activated in AF rabbit/HL-1 cell models, resulting in the upregulation of key downstream protein; this effect was significantly restored by eplerenone. Eplerenone prevented the alterations in circulating neurohormone, reduced the mRNA level of sympathetic and parasympathetic-related growth factors, and inhibited the inducibility and duration of AF.

**Conclusions:** Eplerenone inhibited atrial autonomic nerve remodeling and the occurrence of AF through modulating the ERK1/2 MAPK pathway.

## 1. Introduction

Atrial fibrillation (AF) represents the most common arrhythmia worldwide, which is related to an increased risk of ischemic stroke or systemic embolism [1, 2]. The initiation and progression of AF result from atrial remodeling, including electrical, structural, and contractile remodeling, and it has been shown to contribute continuously to the self-perpetuating nature of AF [3]. However, the detailed mechanisms of the autonomic nervous system (ANS) on AF are not fully understood.

The cardiac ANS is considered to be an important mechanism for the occurrence and maintenance of AF, and previous studies have suggested that the cardiac ANS plays an important role in the process of “AF triggering AF” [4]. Accumulating evidence suggests sympathetic overdistribution in AF patients, with the evidences of increased sympathetic discharge in persistent patients and in animal models [5–7]. Our previous work had also found extensive sympathetic and vagal sprouting in both AF and OSA canine models [8–10]. But to date, the exact mechanisms underlying atrial autonomic remodeling are unknown.

Mitogen-activated protein kinase (MAPK) can increase the risk of AF [11]. The main members of the MAPK superfamily are p38MAPK, extracellular signal-regulated kinase1/2 (ERK1/2), and c-Jun N-terminal kinases (JNKs). Previous studies have shown that the ERK1/2 pathway is related to the autonomic nervous activity [12]. However, it is unclear whether ERK1/2 is associated with autonomic nerve remodeling and further contributes to AF.

Recent studies suggest that elevated aldosterone (ALD) may lead to atrial fibrosis and contribute to AF [13]. Studies in canine models have shown that ALD blockers can suppress fibrosis formation and are thought to prevent AF [14, 15]. Eplerenone is a selective ALD receptor blocker that has shown therapeutic value in the prevention of cardiovascular disease [16]. However, the effects of eplerenone on the atrial nervous remodeling associated with AF are not completely understood. Thus, our study was designed to investigate whether eplerenone prevented atrial nervous remodeling in AF through the ERK1/2 MAPK pathway.

## 2. Materials and Methods

### 2.1. Rabbit AF Model

Then, 21 New Zealand white rabbits (2.5–3.0 kg, Experimental Animal Center of the First Affiliated Hospital of Harbin Medical University, Harbin, China) were randomly divided into sham groups, AF groups, and eplerenone groups. The AF model was established according to our previous studies [17]. The rabbits were anesthetized with ketamine (30–35 mg/kg; Sigma Aldrich, St Louis, MO, United States) and xylazine (5 mg/kg, Sigma Aldrich). After anesthesia, all rabbits' procedures were performed by sterile thoracotomy under mechanical ventilation. A bipolar electrode was implanted in the right atrium and the other side connected to a pacemaker (Fudan University, Shanghai, China), which was placed in a subcutaneous pocket on the backs of rabbits. After 1 week of recovery, the pacemaker began to work at 600 beats/min for 3 weeks. The electrocardiogram (ECG) was randomly checked during the pacemaker operation to ensure that the pacemaker was working. At the end of the experimental procedure, the hearts were rapidly removed after the rabbits were anesthetized.

Rabbits in the AF group were given right atrial pacing for 3 weeks. Rabbits in the eplerenone treatment group were given eplerenone oral (Pfizer, United States) at a dose of 50 mg/day for 28 days, with right atrial pacing starting from the eighth day.

### 2.2. Rat AF Model

Then, 18 Sprague–Dawley rats (8-week-old, 120–150 mg) were randomly divided into three groups: (1) control group: polyethylene glycol (PEG) was pumped for 4 weeks through osmotic minipumps (ALZET pump 2ML, Durect Corporation, Cupertino, CA, United States) implanted subcutaneously; (2) ALD2: ALD (Sigma-Aldrich) was pumped for 2 weeks; and (3) ALD was pumped for 4 weeks. The AF model was established according to previous studies [18]. The rate of ALD release was 1.5 *μ*g/h.

### 2.3. Cell Culture

To determine that MAPK may play a critical role in the neural remodeling of AF, we utilized a well-established HL-1 cell model of AF. HL-1 cells were cultured in flasks in Claycomb medium (JRH Biosciences, Lenexa, KA, United States) supplemented with 10% fetal calf serum, 100 U/mL penicillin, and 100 *μ*L streptomycin (Gibco-BRL, Rockville, MD, United States). HL-1 cells (≥1 × 10^6^ myocytes) were cultured in 6-well plates and subjected to tachypacing by applying a YC-2 stimulator (Chengdu, China), as described in previous studies [17].

### 2.4. Biochemical Measurements

Three sets of blood samples were collected from the hearts after 3 weeks of rapid atrial pacing. They were centrifuged at 3500 × g for 15 min. Norepinephrine (NE), acetyl choline (ACH), and ALD concentration measurement kits were purchased from Jiancheng Biological Technical Institute (China). All procedures followed the manufacturer's instructions.

### 2.5. Atrial Electrophysiological

#### 2.5.1. Rabbit AF Model

The electrophysiological measurements should be done before blood samples are collected. The method was described in our previous study [17]. After anesthetization, intubation, and mechanical ventilation, left side thoracotomy was performed. A four-electrode was sutured to the free wall of the right atrial appendage. The tail end of the electrode was connected with Prucka32 lead electrophysiologic recorder to synchronously record the body surface and intracardiac electrocardiogram. AF was induced by burst stimulation applied to the right atrium with 10 Hz, 2-ms stimuli for 1–10 s. If AF lasts for 30 s, synchronous direct current cardioversion is performed. The duration of AF is recorded for 30 s, and the electrophysiological test is continued after 1 min.

#### 2.5.2. Rat AF Model

AF was induced with essentially the same protocol as described previously in detail [18]. Rats were anesthetized with 1% sodium pentobarbital (30 mg/kg) through peritoneal injection. After open-chest surgery, a 1.9-Foctapolar catheter (Transonic Systems Inc., New York, United States) was placed in the right atrium to deliver programed stimuli. To assess AF inducibility, 50-Hz burst pacing was applied for 3 s with 12 bursts separated by a 2-s interval. AF was defined as > 1 s of irregular atrial electrograms (> 800 beats/min) with an irregular ventricular response. AF duration was defined as the mean duration of all AF episodes within 60 s in each rat.

### 2.6. Immunohistochemical Determination

The paraffin sections of rat and rabbit atrial tissue were stained with hematoxylin and eosin (HE) and Masson, and the atrial fibrosis was observed under a light microscope. The collagen volume fraction () was calculated as the area occupied by collagens divided by the total area in the visual field using Image-Pro Plus software.

Atria tissues were dissected and incubated with antigrowth-associated protein 43 (GAP-43, Abcam), antityrosine hydroxylase (TH, Abcam), antinerve growth factor (NGF, Abcam), and anticholine acetyltransferase (ChAT, Abcam) overnight at 4°C. Then, they were visualized with a DAB-based colorimetric method. The expression of the target proteins was calculated by the digital medical image analysis system (HPISA-1000,Olympus, Shinjuku, Japan).

### 2.7. Real-Time Quantitative Polymerase Chain Reaction

Animal atrial tissue was homogenized in RLT buffer, total ribonucleic acid was extracted using an RNeasy mini kit (Qiagen, Germantown, Maryland), and qRT-PCR was performed using an Applied Bio-system. The primers are shown in Table 1. The relative quantification of gene expression was performed using the 2 − *ΔΔ*CT method after normalization with GAPDH.

### 2.8. Western Blotting

Protein samples were extracted from rabbit atria tissue specimens or HL-1 cells. The protein content was determined by using the Bradford method. The same amount of protein (40 *μ*g) was loaded per lane and separated using denaturing 10% polyacrylamide gels and then transferred moist to polyvinylidene difluoride membranes. Membranes were blocked by 5% nonfat dry milk in PBS and incubated overnight at 4°C. The membranes were probed with antibodies to ChAT (1:500, Abcam), to GAP-43 (1:500, Abcam), to Pan-actin (1:1000, Sigma), and to ERK1/2 (1:500, CST). Bands were visualized using the Super-Signal West Femto Chemiluminescent Substrate (Thermo Scientific, Waltham, MA, United States) and quantified by scanning densitometry (Chemi-DOC, Bio-Rad, Laboratories, Hercules, CA, United States).

### 2.9. Data and Statistical Analyses

The statistical analyses were performed with GraphPad Prism 9.0 software (GraphPad Software, Inc, La Jolla, CA). Continuous variables were presented as the mean ± standard error of mean (SEM). Variables with more than two groups were analyzed by one-way ANOVA followed by Tukey tests. Categorical variables were represented as numbers and percentages and were compared using Chi-square test or Fisher's exact test. Differences were considered statistically significant at *p* < 0.05.

## 3. Results

### 3.1. Eplerenone Inhibited the Rapid Pacing–Induced Atrial Remodeling

**Figure 1:**
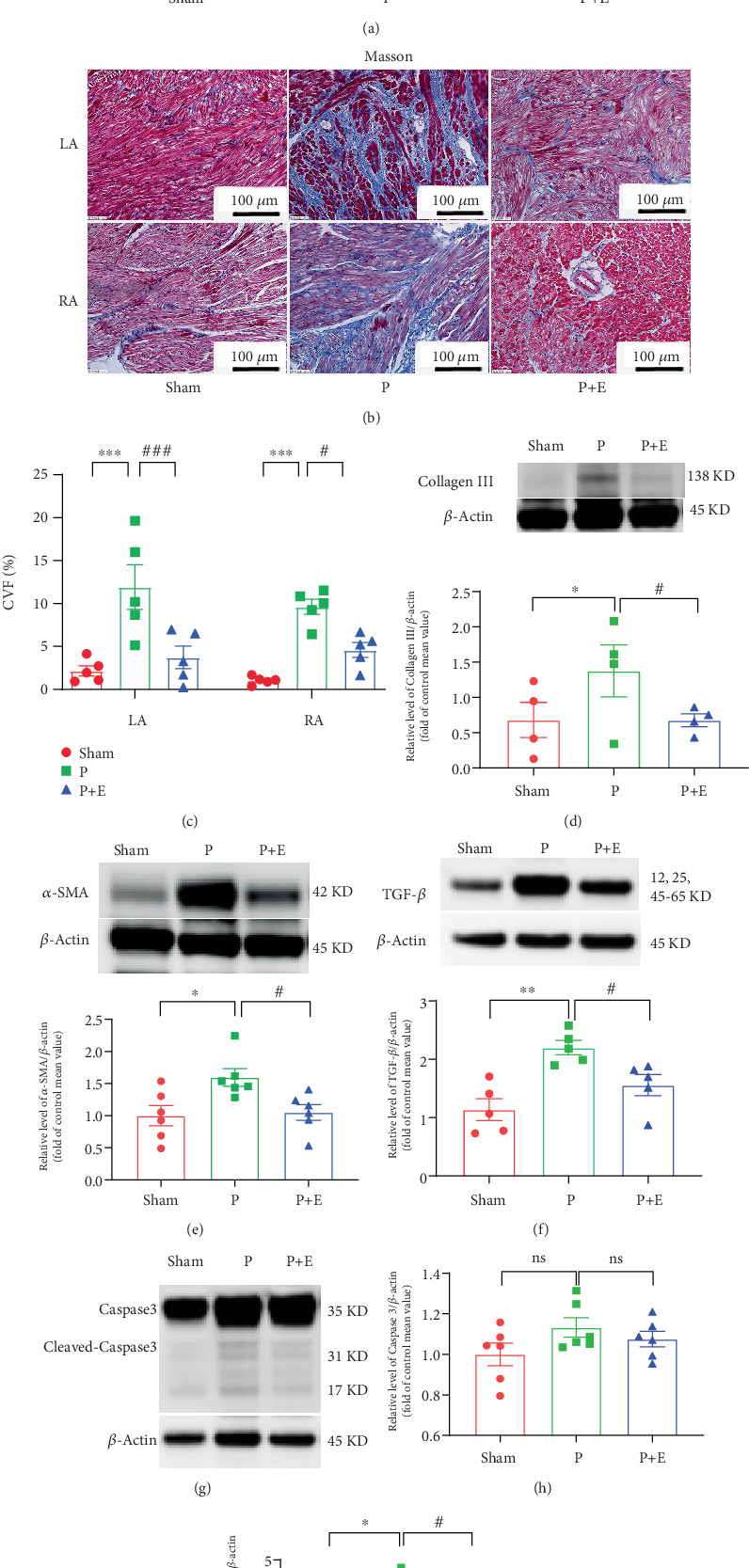
Eplerenone inhibited the rapid pacing-induced atrial remodeling. P = AF group and P + E = AF + eplerenone. (a) HE stains in the left and right atrial myocardium of the groups (20× magnification). (b) Masson's stain in the left and right atrial myocardium of the three groups (20× magnification). (c) Collagen volume fraction (CVF %) of left and right atria in each group. Expressions of (d) collagen III, (e) *α*-SAM, (f) TGF-*β*, and (g–i) Caspase 3. ⁣^∗∗∗^*p* < 0.001 vs. the sham group and ^#^*p* < 0.05 and ^###^*p* < 0.001 vs. the P group; *n* = 6 per group.

HE staining analysis showed that atrial myocytes were neatly arranged, with aligned nuclei and only a small amount of stroma around in the sham operation group. Conversely, the arrangement of cardiomyocytes in the AF group was obviously disordered, loose, or broken, the nuclei were deformed, the connective tissue was hyperplastic, and the distance between cardiomyocytes was obviously enlarged. Eplerenone treatment alleviated these changes (Figure 1a). As shown in Figure 1b, RAP exacerbated interstitial fibrosis in the atria. CVF was significantly higher in the AF group than in the sham group, which was reversed by eplerenone (Figure 1c). Fibrosis-related proteins including collagen III (Figure 1d), *α*-SAM (Figure 1e), and TGF-*β* (Figure 1f) were significantly increased in the RAP group; however, eplerenone could ameliorate these changes. The apoptosis-related proteins cleaved-caspase3 (Figures 1g, 1h, and 1i) were significantly increased in the RAP group, while eplerenone could ameliorate these changes.

### 3.2. Rapid Atrial Pacing and Excessive Circulating ALD Can Increase AF Susceptibility, While Eplerenone Attenuated AF Promotion

**Figure 2:**
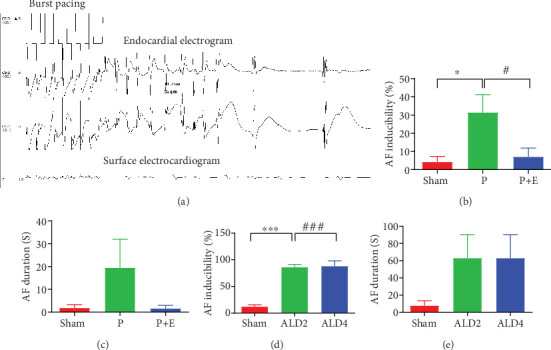
Rapid atrial pacing and excessive circulating ALD can increase AF susceptibility, while eplerenone attenuated AF promotion. (a) AF was induced after rapid pacing. (b) AF inducibility in rabbit models (*n* = 7). (c) AF duration in rabbit models (*n* = 7). (d) AF inducibility in rat models (*n* = 5). (e) AF duration in rat models (*n* = 5). ALD2 = aldosterone was pumped for 2 weeks; ALD4 = aldosterone was pumped for 4 weeks; ⁣^∗^*p* < 0.05 versus the sham group and ^#^*p* < 0.05 versus the AF group.

In the right atrial rapid pacing rabbit model, AF inducibility was significantly increased in the AF group compared with the sham group at baseline. However, compared to the AF group, the incidences of AF were markedly reduced in the eplerenone treatment group (Figure 2b). Similar to AF inducibility, AF duration was increased in the AF groups, which was reduced by eplerenone (Figure 2c). In the rat AF model, AF inducibility was significantly increased in the ALD2 and ALD4 groups compared with the sham group (Figure 2d), and AF duration was also prolonged in ALD2 and ALD4 (Figure 2e).

### 3.3. The Effects of Eplerenone on the ANS Activity in Rabbit AF Models

**Figure 3:**
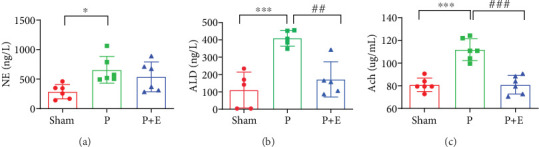
The effects of eplerenone on the autonomic nervous system activity in rabbit AF models. (a) Serum NE concentration in each group. (b) Serum ALD concentration in each group. (c) Serum ACH concentration in each group. ⁣^∗^*p* < 0.05 and ⁣^∗∗∗^*p* < 0.001 vs. the sham group and ^##^*p* < 0.01 and ^###^*p* < 0.001 vs. the P group; *n* = 6 per group.

Sera NE, ACH, and ALD concentrations are important indicators of parasympathetic and sympathetic activity. The blood was collected from the hearts, and NE, ACH, and ALD were detected after electrophysiological tests in rabbit models. As shown in Figure 3a, serum NE was distinctly increased in the AF group compared with the sham group; however, eplerenone did not significantly inhibit this change (Figure 3a). Sera ALD and ACH concentrations were significantly increased in the rabbit model of AF, which eplerenone inhibited the changes (Figure 3b,c).

### 3.4. The Effects of Eplerenone on mRNA Expressions of Cx40, Cx43, M2 Receptor, *β*1 Receptor, and *β*2 Receptor in Rabbit AF Models

**Figure 4:**
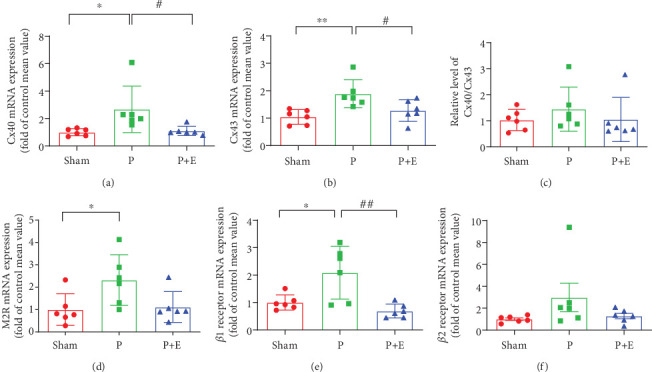
The effects of eplerenone on mRNA expression of Cx40, Cx43, M2 receptor, *β*1 receptor, and *β*2 receptor in rabbit AF models. (a) The mRNA expression of Cx40 in atrial tissue. (b) The mRNA expression of Cx43 in atrial tissue. (c) The ratio of Cx40/Cx43. (d) The mRNA expression of M2 receptor in atrial tissue. (e) The mRNA expression of *β*1 receptor in atrial tissue. (f) The mRNA expression of *β*2 receptor in atrial tissue. ⁣^∗^*p* < 0.05 and ⁣^∗∗^*p* < 0.01 vs. the sham group and ^#^*p* < 0.05 and ^##^*p* < 0.01 vs. the P group; *n* = 6 per group.

Both the expressions of Cx40 and Cx43 were significantly increased in AF atria compared with the sham group, which was reversed by eplerenone in the treatment group (Figure 4a,b). These findings indicated that the gap junction was impaired after atrial rapid pacing for 3 weeks and that the damage could be reversed by eplerenone.

The mRNA levels of *β*1, *β*2, and M2 receptors were increased in the AF group compared with the sham group. However, eplerenone has been shown to significantly reverse the upregulation of *β*1 receptor and slightly attenuate the upregulation of *β*2 and M2 receptor genes (Figures 4d, 4e, and 4f).

### 3.5. The Effects of Eplerenone on Protein Expressions of ChAT, GAP-43, and ERK1/2 in Rabbit AF Models

**Figure 5:**
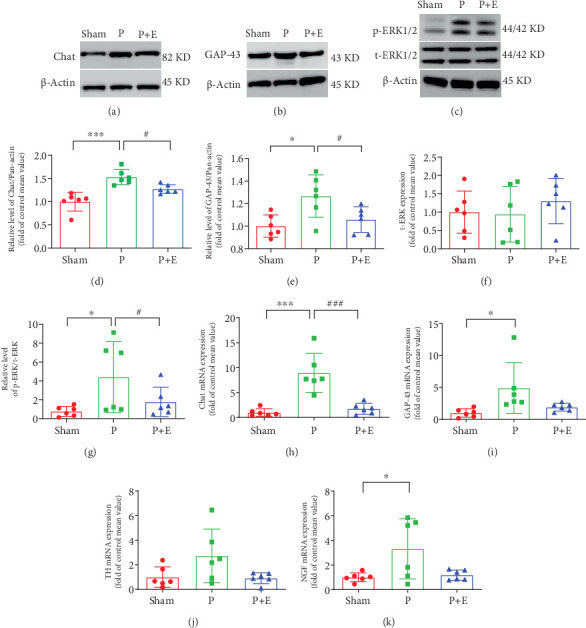
The effects of eplerenone on protein expression of ChAT, GAP-43, and ERK1/2 in rabbit AF models. (a–g) Representative immunoblots of ChAT, GAP-43, and ERK1/2 for atrial tissue samples in each group. (h) ChAT mRNA expression in left and right atria. (i) GAP-43 mRNA expression in left and right atria. (j) TH mRNA expression in left and right atria. (k) NGF mRNA expression in left and right atria. ⁣^∗^*p* < 0.05 and ⁣^∗∗∗^*p* < 0.001 vs. the sham group, ^#^*p* < 0.05 and^###^*p* < 0.001 vs. the P group; *n* = 6 each group.

The expression of ERK1/2, ChAT, and GAP-43 protein was significantly upregulated in AF atrial tissue compared with the sham group at baseline, which was reversed by eplerenone (Figures 5a, 5b, 5c, 5d, 5e, and 5g).

### 3.6. The Effects of Eplerenone on Autonomic Nerve Remodeling in Rabbit and Rat AF Models

**Figure 6:**
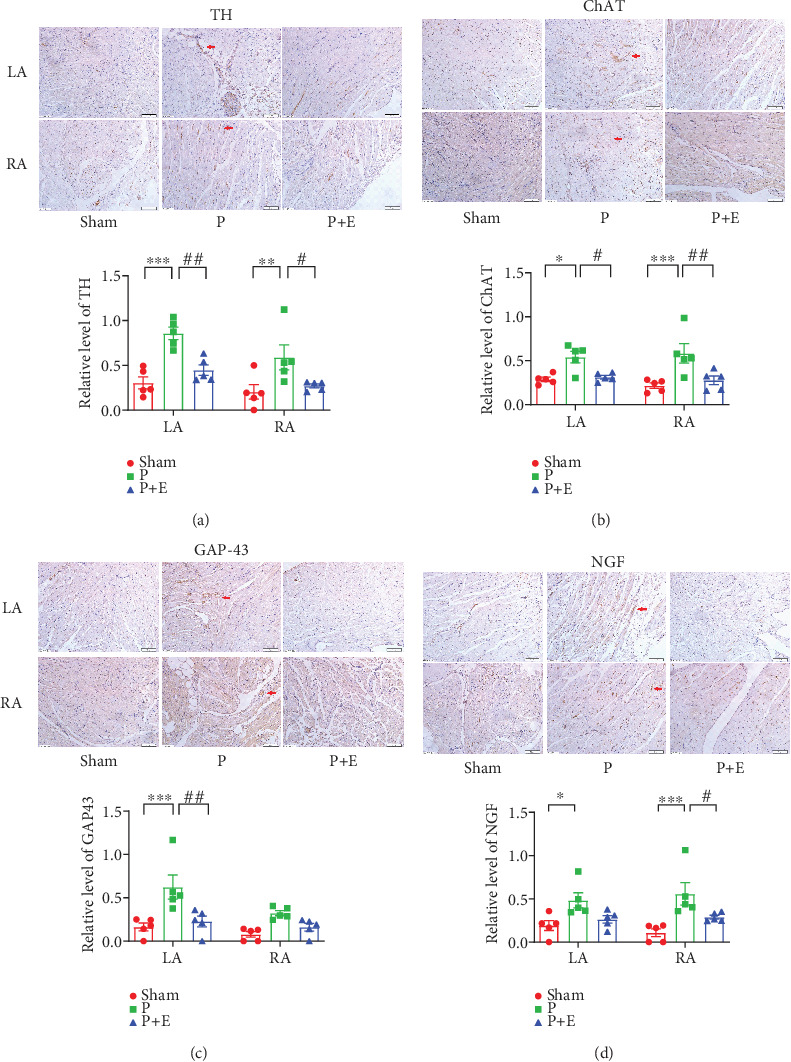
Sympathetic and parasympathetic sprouting and distribution of nerve fibers in rabbit AF models. (a) Tyrosine hydroxylase (TH) expression in left and right atria. (b) Choline acetyltransferase (ChAT) expression in left and right atria. (c) Growth-associated protein 43 (GAP-43) expression in left and right atria. (d) Nerve growth factor (NGF) expression in left and right atria. The magnification is × 20. ⁣^∗^*p* < 0.05, ⁣^∗∗^*p* < 0.01, and ⁣^∗∗∗^*p* < 0.001 vs. the sham group and ^#^*p* < 0.05, ^##^*p* < 0.01, and ^###^*p* < 0.001 vs. the P group; *n* = 5 each group.

**Figure 7:**
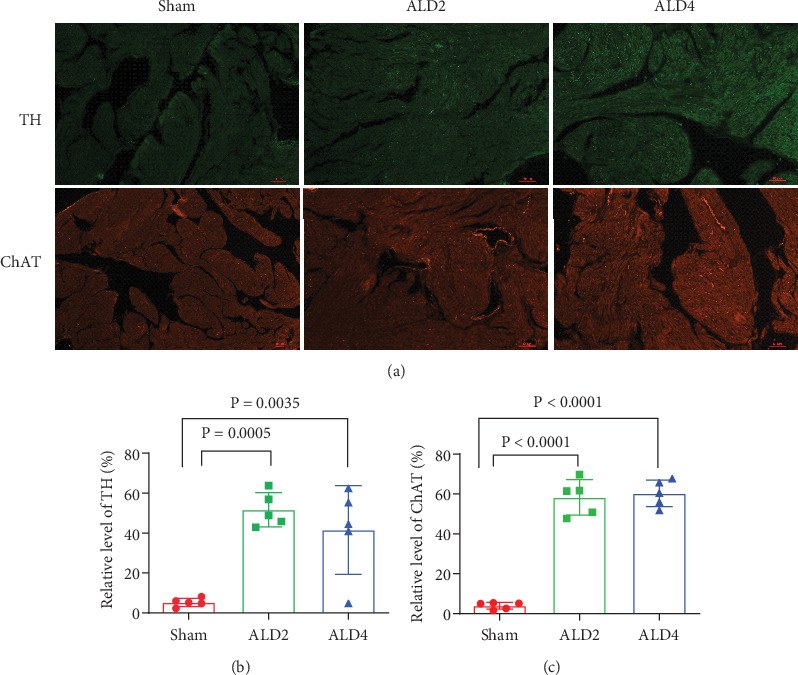
Immunofluorescent staining of TH and ChAT in rat models. (a) Representative images of tyrosine hydroxylase (TH) and choline acetyltransferase (ChAT) expression in rat atria. (b) Statistical results for expression of TH in rat atria. (c) Statistical results for expression of ChAT in rat atria. Green represents TH staining; red represents ChAT staining; ALD2 = aldosterone was pumped for 2 weeks; ALD4 = aldosterone was pumped for 4 weeks; *n* = 5 each group.

ANS remodeling in the atria of rabbits and rats was further examined. We detected the mRNA expressions of ChAT, GAP-43, TH, and NGF. The expressions of the ChAT, GAP-43, and NGF genes were obviously increased in the atria of the AF group compared with the sham group. But, eplerenone alleviated these changes (Figures 5h, 5i, and 5k). In addition, the TH, GAP-43, NGF, and ChAT positive fiber density was higher in both the left and right atria of AF rabbits than that in sham rabbits. However, eplerenone treatment can reverse the changes (Figure 6). In rat AF models, we also found that the immunofluorescent staining of TH and ChAT in rat atria significantly increased in both the ALD2 and ALD4 groups (Figure 7).

### 3.7. ERK1/2 MAPK Mediated the Overexpression of GAP-43 and ChAT in HL-1 Cells

**Figure 8:**
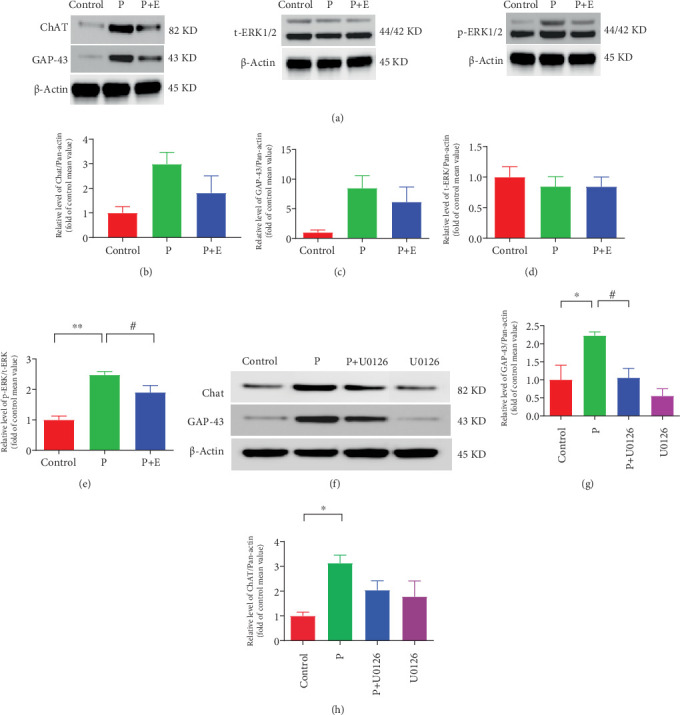
Effects of eplerenone and ERK1/2 inhibitors on the expressions of ChAt, GAP-43, t-ERK1/2, and p-ERK/t-ERK in HL-1 cardiomyocytes. (a) Representative Western blots of ChAt, GAP-43, and ERK1/2 for HL-1 cardiomyocyte samples in each group. (b) Protein expressions of ChAt in the three groups. (c) Protein expressions of GAP-43 in the three groups. (d) Protein expressions of t-ERK in the three groups. (e) Protein expressions of p-ERK in the three groups. (f) Representative Western blots of ChAt and GAP-43 for HL-1 cardiomyocyte samples in the four groups. (g) Protein expressions of GAP-43 in the four groups. (h) Protein expressions of ChAt in the four groups. Data are expressed as mean ± SEM. ⁣^∗^*p* < 0.05 and ⁣^∗∗^*p* < 0.01 vs. the control group and ^#^*p* < 0.05 vs. the P group; *n* = 3 each group.

Consistent with animal experiment data, the protein expression of p-ERK1/2 in HL-1 cells were obviously increased in the pacing group, which was attenuated by eplerenone treatment (Figure 8a,e). In addition, inhibitors of ERK1/2 significantly suppressed the upregulation of GAP-43 (Figure 8f,g) or moderately suppressed the upregulation of ChAT protein (Figure 8h).

## 4. Discussion

### 4.1. Main Finding

We have demonstrated that significant parasympathetic and sympathetic nerve remodeling was present in vitro and in vivo in the AF model. Eplerenone markedly suppressed atrial autonomic nerve remodeling by inhibiting the ERK1/2 MAPK signaling pathway.

### 4.2. Excess ALD Levels Are Involved in Atrial Structural Remodeling in AF

Atrial structural remodeling is characterized by interstitial fibrosis [19]. It is known that ALD is closely related to atrial fibrosis and contributes to human AF according to previous clinical studies [20]. Experimental results also suggest that ALD may cause a substrate for atrial fibrosis and AF [21]. ALD can lead to the upregulation of profibrotic mediators and collagen synthesis, which is prevented by mineralocorticoid receptor blockers [21]. Our findings are consistent with the previous clinical and animal studies. Serum ALD concentration was obviously increased in the AF group. Masson's staining showed a marked increase in CVF, which indicated that atrial fibrosis increased significantly after 3 weeks of rapid pacing. However, these changes could be attenuated by eplerenone. Furthermore, Cx40 and Cx43 were observed to be significantly increased in the AF group, which indicated that the gap junction was impaired after atrial rapid pacing and led to abnormal transmission and AF [22].

### 4.3. Atrial ANS Remodeling and AF

Atrial autonomic remodeling may provide an important substrate for AF, and both sympathetic and parasympathetic activation differently influence atria electrophysiology [23]. Previous studies had found that atrial autonomic remodeling occurs in AF animal models [24, 25]. In the present study, we also found that autonomic nerve sprout was significantly increased in RAP rabbits, which was markedly attenuated by eplerenone treatment. Consequently, we hypothesize that eplerenone may reduce the incidence of AF by inhibiting atrial autonomic neural remodeling. In order to elucidate the specific mechanism of autonomic nerve remodeling in AF, we investigated, respectively, the effects of sympathetic and parasympathetic nerves on AF.

In our study, we found that the mRNA or protein expression of ChAT significantly increased both in vivo and in vitro in AF models, which can be inhibited by eplerenone or ERK1/2 inhibitors. As one of the specific markers of cholinergic neurons, ChAT is synthesized in the soma of cholinergic neurons, transported to the nerve endings, and finally released [26]. Cholinergic M2 receptors are the primary mediators of parasympathetic control of heart function. ACH binds to muscarinic cholinergic receptors and activates the ACH-regulated potassium current (*I*_K−ACH_), which mediates shorting of ERP [27]. Shortened ERP can significantly increase the incidence of AF. In the present study, we found that M2R was upregulated in RAP rabbits, which indicated that the upregulation of M2R participated in parasympathetic remodeling in RAP models and led to an increased susceptibility to AF. In addition, we also found that sera ACH and ALD concentrations were significantly increased in AF models. These findings hint that the vagal nerve may play an important role in the initiation and maintenance of AF. However, eplerenone could reverse these changes.

Although sympathetic seems to have a less prominent effect on the atrial electrophysiological parameters in normal subjects, it is more crucial for the genesis of AF in the remodeled atria [28]. We conducted TH immunohistochemical staining on atrial tissue samples obtained from rabbits with AF and observed that TH-positive fiber density was higher in both left and right atria of AF rabbits than that in sham rabbits. Furthermore, we quantified the mRNA levels of sympathetic hormone receptors in atrial tissue. Our results demonstrated a significant increase in *β*1 receptor density in the atrial tissue of the AF group compared to the control group. In contrast, only a marginal increase was observed in *β*2 receptors, which did not reach statistical significance. This discrepancy can be attributed to the predominant distribution of *β*1 receptors in cardiac tissue, whereas *β*2 receptors are primarily localized in vascular and bronchial tissues.

GAP-43 and NGF were the markers of nerve sprouting; they had been shown to be a key stimulus underlying the sprouting of both sympathetic and parasympathetic nerves in the heart. It has been reported to increase in atrial tissue in an animal model of AF and also in the human AF atrium [29–31]. In the present study, both the expressions of NGF and GAP-43 and the nerve staining with NGF and GAP-43 were elevated in the atria of the AF group.

Recently, a growing body of research emphasizes the importance of autonomic imbalance in AF. Combined firing of sympathetic and parasympathetic nerves has been demonstrated immediately prior to the onset of atrial arrhythmias in animal models of AF [25, 32], as well as in clinical studies [33]. Our findings are consistent with previous research. Parasympathetic and sympathetic activity was detected and we found that the increase of sera ALD and ACH concentrations was significantly higher than NE in AF models, whereas the autonomic imbalance was reversed by eplerenone. It follows that both adrenergic and cholinergic nerves were likely activated during AF. This ultimately caused an imbalance in the automatic nervous system, and this simultaneous activation of the adrenergic and cholinergic nerves resulted in arrhythmia.

### 4.4. ERK1/2 MAPK Pathway Participates in Atrial ANS Remodeling in AF

In our study, we observed a significant upregulation of ERK1/2 protein expression in the atrial tissue of AF rabbits. Treatment with eplerenone markedly attenuated this upregulation. Subsequently, we conducted validation experiments at the cellular level as well. The expression of ERK1/2 was upregulated in paced HL-1 cells, whereas this upregulation was inhibited by eplerenone. In another set of paced HL-1 cells, the upregulation of GAP-43 was inhibited by U0126 (an ERK1/2 blocker). ERK1/2 has been proved to participate in atrial structural remodeling in AF [34]. However, much less is known about the relationship between ERK1/2 and ANS remodeling. Our findings suggest that eplerenone can inhibit atrial autonomic nerve remodeling, possibly through its effects on the ERK1/2 MAPK pathway. Further research is needed to elucidate the exact mechanisms involved.

### 4.5. MRA Plays an Important Role in Preventing ANS Remodeling

Some studies have found that MRA will benefit AF [14, 35]. There are also some meta-analyses to prove that MRA significantly reduces new-onset AF and recurrent AF [36, 37]. In our study, we found that eplerenone played an important role in preventing autonomic nerve remodeling of AF. Our results are in agreement with previous observations and prove the effectiveness of eplerenone in AF both in vivo and in vitro. We also highlighted the possible mechanism behind the phenomenon.

To our knowledge, this is the first study to show that eplerenone can inhibit atrial autonomic nerve remodeling, beyond that it can ameliorate atrial electrical and structural remodeling. Therefore, eplerenone could be regarded as a promising novel upstream therapy for the treatment of AF. We believe that eplerenone will be useful for treating patients with AF in the clinic.

## 5. Conclusion

We demonstrated that the ERK1/2 MAPK pathway participates in atrial autonomic nerve remodeling during AF and that eplerenone inhibited atrial autonomic nerve remodeling by regulating the ERK1/2 MAPK pathway. Our study provides novel insights into the pharmacological role of eplerenone against AF.

## Figures and Tables

**Table 1:** Primers.

ChAT	Forward	5⁣′-AGGCCATCGTGCAGCGTTTT-3⁣′	139 (bp)
ChAT	Reverse	5⁣′-GCCAGGCGGTTATTGAGGTA-3⁣′	
M2 receptor	Forward	5⁣′-GACACTAAGGGACCCGAGAT-3⁣′	237 (bp)
M2 receptor	Reverse	5⁣′-GTCAGGACCTTTCTGCTTCA-3⁣′	
NGF	Forward	5⁣′-CACTCTAACGAAGTTCTGGC-3⁣′	157 (bp)
NGF	Reverse	5⁣′-ACATGGACATTACGCTATGC-3⁣′	
GAP43	Forward	5⁣′-GCGAGGCTGACCAAGAACAT-3⁣′	135 (bp)
GAP43	Reverse	5⁣′-ACGTGAGCAGGACAGGAAGG-3⁣′	
TH	Forward	5⁣′-GCTACAGCGAGGACCACATC-3⁣′	184 (bp)
TH	Reverse	5⁣′-GGCGAGTGCATCGTTGAG-3⁣′	
CX40	Forward	5⁣′-AAGCCTTCCCCATCTCCCACA-3⁣′	150 (bp)
CX40	Reverse	5⁣′-GCACCTCCTTGCCCGTCTCG-3⁣′	
CX43	Forward	5⁣′-TCAGCCTGAGTGCCGTTTAC-3⁣′	124 (bp)
CX43	Reverse	5⁣′-GACACCAACGACACCACCAG-3⁣′	
*β*1 receptor	Forward	5⁣′-AGAATGTCACCAACCGTAGC-3⁣′	288 (bp)
*β*1 receptor	Reverse	5⁣′-GAGGTCCATGAGGTAGTAGAGG-3⁣′	
*β*2 receptor	Forward	5⁣′-ATGTATCTGGGTTTCATCCG-3⁣′	176 (bp)
*β*2 receptor	Reverse	5⁣′-CCATTTCACTGTCATAGGCT-3⁣′	
GAPDH	Forward	5⁣′-GGGAAACTCACTGGCATGG-3⁣′	209 (bp)
GAPDH	Reverse	5⁣′-CACTGTTGAAGTCGCAGGAGA-3⁣′	

## Data Availability

The original contributions presented in the study are included in the article material; further inquiries can be directed to the corresponding author.
